# MonoAux: Fully Exploiting Auxiliary Information and Uncertainty for Monocular 3D Object Detection

**DOI:** 10.34133/cbsystems.0097

**Published:** 2024-03-27

**Authors:** Zhenglin Li, Wenbo Zheng, Le Yang, Liyan Ma, Yang Zhou, Yan Peng

**Affiliations:** ^1^ Institute of Artificial Intelligence, Shanghai University, Shanghai, China.; ^2^ School of Future Technology, Shanghai University, Shanghai, China.; ^3^ Department of Electrical and Computer Engineering, University of Canterbury, Christchurch, New Zealand.

## Abstract

Monocular 3D object detection plays a pivotal role in autonomous driving, presenting a formidable challenge by requiring the precise localization of 3D objects within a single image, devoid of depth information. Most existing methods in this domain fall short of harnessing the limited information available in monocular 3D detection tasks. They typically provide only a single detection outcome, omitting essential uncertainty analysis and result post-processing during model inference, thus limiting overall model performance. In this paper, we propose a comprehensive framework that maximizes information extraction from monocular images while encompassing diverse depth estimation and incorporating uncertainty analysis. Specifically, we mine additional information intrinsic to the monocular 3D detection task to augment supervision, thereby addressing the information scarcity challenge. Moreover, our framework handles depth estimation by recovering multiple sets of depth values from calculated visual heights. The final depth estimate and 3D confidence are determined through an uncertainty fusion process, effectively reducing inference errors. Furthermore, to address task weight allocation in multi-task training, we present a versatile training strategy tailored to monocular 3D detection. This approach leverages measurement indicators to monitor task progress, adaptively adjusting loss weights for different tasks. Experimental results on the KITTI and Waymo dataset confirm the effectiveness of our approach. The proposed method consistently provides enhanced performance across various difficulty levels compared to the original framework while maintaining real-time efficiency.

## Introduction

In autonomous driving, vehicles require a continuous and robust understanding of their environment to ensure safe operation. This primarily involves acquiring information about the distribution of obstacles. Within this context, target detection plays a pivotal role as it enables the simultaneous determination of object category, location, and size within the surrounding environment [1–3]. These information serves as a vital foundation for the downstream autonomous driving modules. The advent of deep learning has led to notable advancements in two-dimensional (2D) target detection tasks [4–6]. Nevertheless, tasks like autonomous driving, which necessitate comprehensive 3D scene information, pose extra challenges. They stem from factors such as variations in the environment, object postures, and occlusions. Consequently, the information provided by 2D target detection alone cannot meet the stringent requirements for ensuring safe autonomous driving. Building upon these considerations, 3D object detection has gained increased attention. Modeling object detection results in the form of 3D bounding boxes offers a more comprehensive representation of spatial information, enabling vehicles to perceive their surroundings more accurately. This, in turn, supports various downstream tasks critical for autonomous driving safety. It is worth noting that some 3D object detection methods rely on expensive Light Detection and Ranging(LiDAR) sensors or multiple cameras, which significantly raise the implementation complexity and overall system cost [7–10]. In contrast, monocular 3D target detection, characterized by its affordability and computational efficiency, stands out as an essential component of real-time autonomous driving systems. Its lightweight nature and cost-effectiveness have gained significant interest and widespread adoption in the autonomous driving community [1,3,11,12].

Monocular 3D target detection aims to recover spatial information, encompassing object position and depth, from a solitary image. The main challenge lies in the precise inference of 3D spatial attributes, particularly depth, in situations where only visual imagery is available. Knowledge of an object’s visual appearance facilitates the deduction of its spatial coordinates, grounded in principles of imaging geometry [13,14]. Therefore, factors such as prior knowledge of the camera’s imaging process and physical dimensions of the object are of paramount importance in ensuring the accuracy of monocular 3D target detection.

In addressing the intricate task of monocular 3D object detection, ongoing research proposes diverse solutions. For instance, [15–18] harness global or local geometric relationships implicit in 2D images to confine the detection of 3D targets, thereby furnishing enhanced geometric priors. [19,20] employ principles of camera imaging to calculate distances based on physical and projected visual heights, thereby ameliorating the issue of inaccurate depth estimation. However, despite their contributions in addressing the insufficiency of effective information in monocular 3D target detection, these methods exhibit certain limitations: (a) incomplete utilization of the information encapsulated in monocular 3D detection tasks, particularly the presence of annotated information within the images, which can serve as a valuable source of supervisory data for training, and (b) predominantly providing a solitary detection outcome, without comprehensive uncertainty analysis and post-processing of the results.

To address the issues outlined above, we propose a monocular 3D target detection framework that maximizes the utilization of information within monocular images, facilitating diverse depth estimation while incorporating measures of uncertainty. First, we capitalize on annotation information within the images to introduce supplementary supervisory cues, encompassing parameters such as the target’s occlusion level, displacement of the eight corner points of the 3D bounding box concerning the projected 2D center, as well as the dimensions of the 2D bounding box. Furthermore, the bird’s eye view (BEV) plane and homographic transformations between 2D image planes are leveraged to provide additional supervision for both 2D and 3D detection tasks. Second, we propose the recovery of diverse depth information from multiple sets of visual heights computed from the corner points of the object bounding box. This approach entails modeling the uncertainty associated with predictions from different branches, ultimately culminating in the derivation of the final depth estimate and 3D confidence through uncertainty fusion. Additionally, we propose a comprehensive training strategy tailored for multi-task learning in monocular 3D detection, aimed at reducing errors in target position and size estimation during the training process.

Experimental findings on the KITTI [21] and Waymo [22] dataset validate the effectiveness of our proposed framework, demonstrating varying degrees of improvement across easy, moderate, and hard difficulty levels compared to the original framework.

The primary contributions of this work are as follows:

(a) We harness the latent information embedded within 3D annotations and leverage the inter-plane transformation relationships to introduce supplementary supervisory branches, effectively compensating for the deficiency in the available information within the monocular 3D detection task.

(b) We propose a novel approach that leverages estimation uncertainty to dynamically fuse multiple sets of depth values and refine 3D confidence, thereby mitigating the impact of errors inherent in a single detection result.

(c) We present a versatile and adaptable training strategy, specifically tailored for multi-task learning in monocular 3D target detection, with the objective of improving the accuracy of target position and size estimation during the training process.

## Related Work

### Monocular 3D object detection

Monocular 3D object detection is the task of predicting 3D bounding boxes from a single input image. This problem is inherently challenging and has traditionally lagged behind LiDAR-based and stereo image-based detection methods [3,23]. To bridge this gap and achieve competitive results, some approaches incorporate supplementary information during the training stage.

MonoPSR [24] explores LiDAR point clouds to reconstruct shape and scale information for 2D region proposals. CaDDN [25] trains a network to predict pixel-level depth distribution using dense depth maps and transforms front view image features into BEV representations for 3D object detection. OccupancyM3D [26] employs synchronized sparse LiDAR points to generate occupancy labels based on CaDDN, introducing corresponding occupancy losses to enable the network to learn voxelized occupancy in frustum and 3D space. MonoPixel [27] utilizes raw LiDAR points to introduce a novel object-centered auxiliary depth loss, enhancing the depth estimation for learned objects.

While these approaches are promising, models incorporating additional auxiliary information often rely on extra modules, leading to increased computational costs. Consequently, they tend to exhibit slower inference speeds, limiting their practical applicability in real-time autonomous driving scenarios. In contrast, some methods focus solely on information contained within the monocular image and improve upon geometric relationships or model frameworks inherent in the image.

MonoJSG [28] introduces semantic and geometric cost volumes to enhance object distance recovery. DID-M3D [15] decomposes an object’s instance depth into visual depth and attribute depth. MonoRCNN [14] and GUPNet [19] recover distance information through physical height and projected visual height. MonoPair [12] considers pairwise relationships between adjacent objects, using spatial constraints for detection optimization. MonoFlex [29] presents a flexible framework for monocular 3D object detection, explicitly addressing truncated objects and adaptively combining multiple depth estimation methods. MonoGround [30] introduces ground plane information as a prior, serving as an additional geometric condition for mapping and an extra source of depth estimation to enhance accuracy.

While many of these methods introduce additional geometric priors, they often ignore object-specific differences. These differences can be affected by objects outside the predicted distribution, potentially leading to performance degradation.

In contrast, our proposed approach explicitly utilizes information present in the monocular image, reducing dependence on additional modules and decreasing computational costs. We propose additional branches to enforce global geometric constraints on the target, facilitating efficient learning. During inference, we obtain multiple sets of diverse depth estimates and adaptively combine them to estimate the final object depth. This approach differs from using a single method for all objects, improving depth estimation accuracy.

### Uncertainty estimation in object detection

Bayesian modeling is a prominent approach in addressing two primary forms of uncertainty in the field [31]. Epistemic uncertainty pertains to the uncertainty associated with model parameters, while aleatoric uncertainty accounts for the inherent observational noise. Gaussian YOLO [32], for instance, incorporates the uncertainty surrounding predicted 2D bounding boxes to refine detection scores. The uncertainty-aware regression loss [31], as a direct modeling approach, effectively rebalances samples within the network and redirects focus toward more reasonable samples, thereby enhancing overall accuracy. The gradient-based method [33] introduces a gradient-based metric for quantifying uncertainty, allowing for the generation of uncertainty information from hidden network layers and its evaluation during post-processing. MonoPair [12] employs uncertainty to assign weights for post-optimization between predicted 3D positions and pairwise constraints. DID-M3D [15] decouples instance depth into attribute depth and visual depth, predicting corresponding uncertainties to express confidence in depth estimation. These various types of depth information are then aggregated to determine the final instance depth.

In our work, we develop a framework to model uncertainty across multiple sets of estimated depths, thereby quantifying their respective contributions to the final prediction outcome.

## Materials and Methods

### Framework overview

**Fig. 1.**
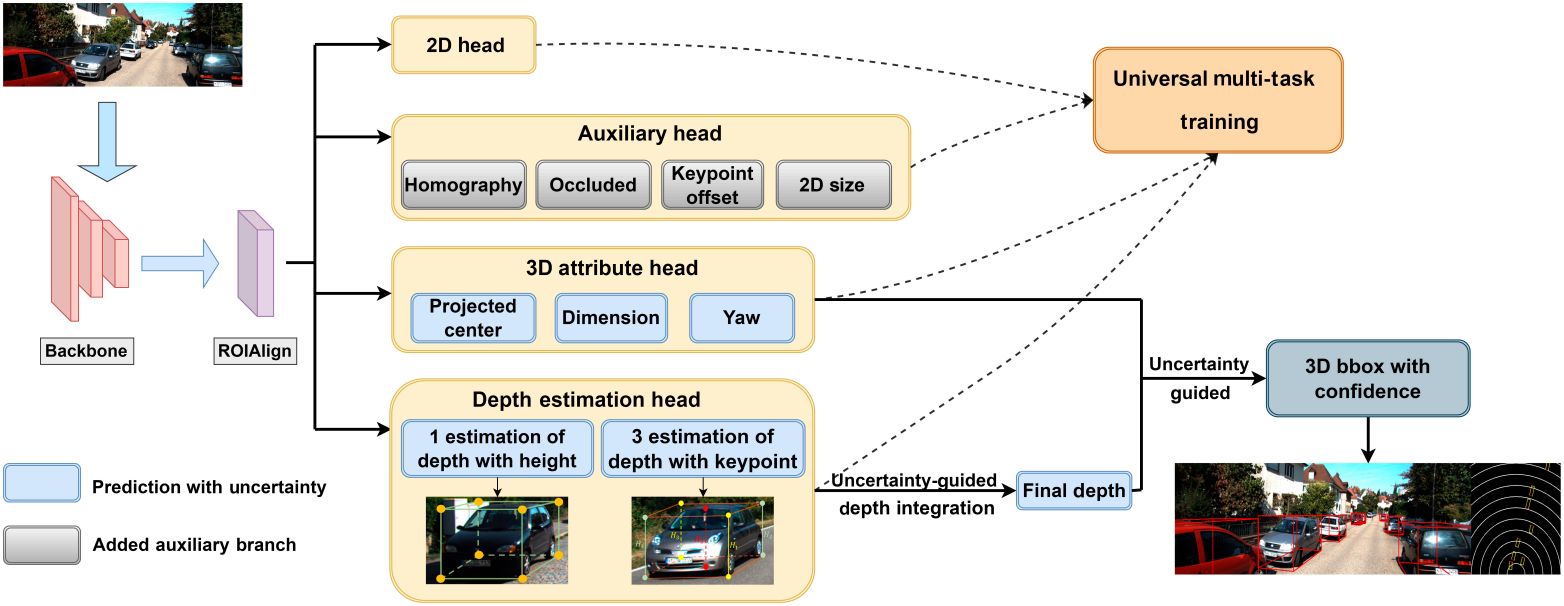
Overview of our framework.

Figure 1 shows the framework of the proposed MonoAux. Initially, a comprehensive set of object features is extracted from the input image. Following that, multiple network heads are instantiated to offer distinct object attributes, including the 2D head, auxiliary head, 3D attribute head, and depth estimation head. Given the specific nature of the monocular 3D target detection task, the inclusion of the auxiliary head plays a pivotal role in constraining the overall objective. The depth estimation head is introduced to yield four distinct sets of depth values, along with associated uncertainty estimates, acquired through both direct estimation and key point analysis. These diverse depth estimates are subsequently fused using an uncertainty-guided integration strategy, yielding the ultimate depth value alongside its corresponding uncertainty.

Furthermore, the uncertainty values pertaining to depth and other 3D attributes are translated into confidence scores, culminating in the derivation of the 3D bounding box with confidence. Finally, the proposed universal multi-task training (UMT) strategy serves as an additional measure to enhance the accuracy of target position and size estimation throughout the training process. UMT oversees the complete training process, resulting in enhanced precision and stability.

### Auxiliary regression heads

Indeed, within the image, there exists a wealth of valuable annotation information pertaining to objects, which presents a rich source of high-quality supervisory data for training purposes. Nevertheless, the majority of contemporary research efforts fall short in harnessing the full potential of the information inherent in monocular 3D detection tasks. We thus propose to add several different auxiliary branches to meet the constraints of additional information. This section introduces each of our proposed auxiliary branches. Note that the auxiliary branches are explicitly employed during the training phase.

#### Homography head

Many contemporary approaches treat individual 3D objects within a scene as isolated training samples, often neglecting their inherent geometric interrelationships [34,35]. This results in underutilization of spatial constraints, necessitating an assumption that the position of a single object is influenced by the surrounding objects. To address this problem, we propose a homography branch aimed at constraining the detection of 3D objects.

The coordinate transformation between 2D points, *p_1_* and *p_2_*, on two different planes can be accomplished using a homography matrix denoted as *M*:

$${\mathrm{sp}}_{1}={\mathrm{Mp}}_{2}$$ (1)

*M* signifies the homography matrix representing the transformation between the two planes and *s* represents the scaling factor. Notably, the homography matrix possesses eight degrees of freedom, mandating the presence of at least four corresponding point pairs to deduce the matrix.

**Fig. 2.**
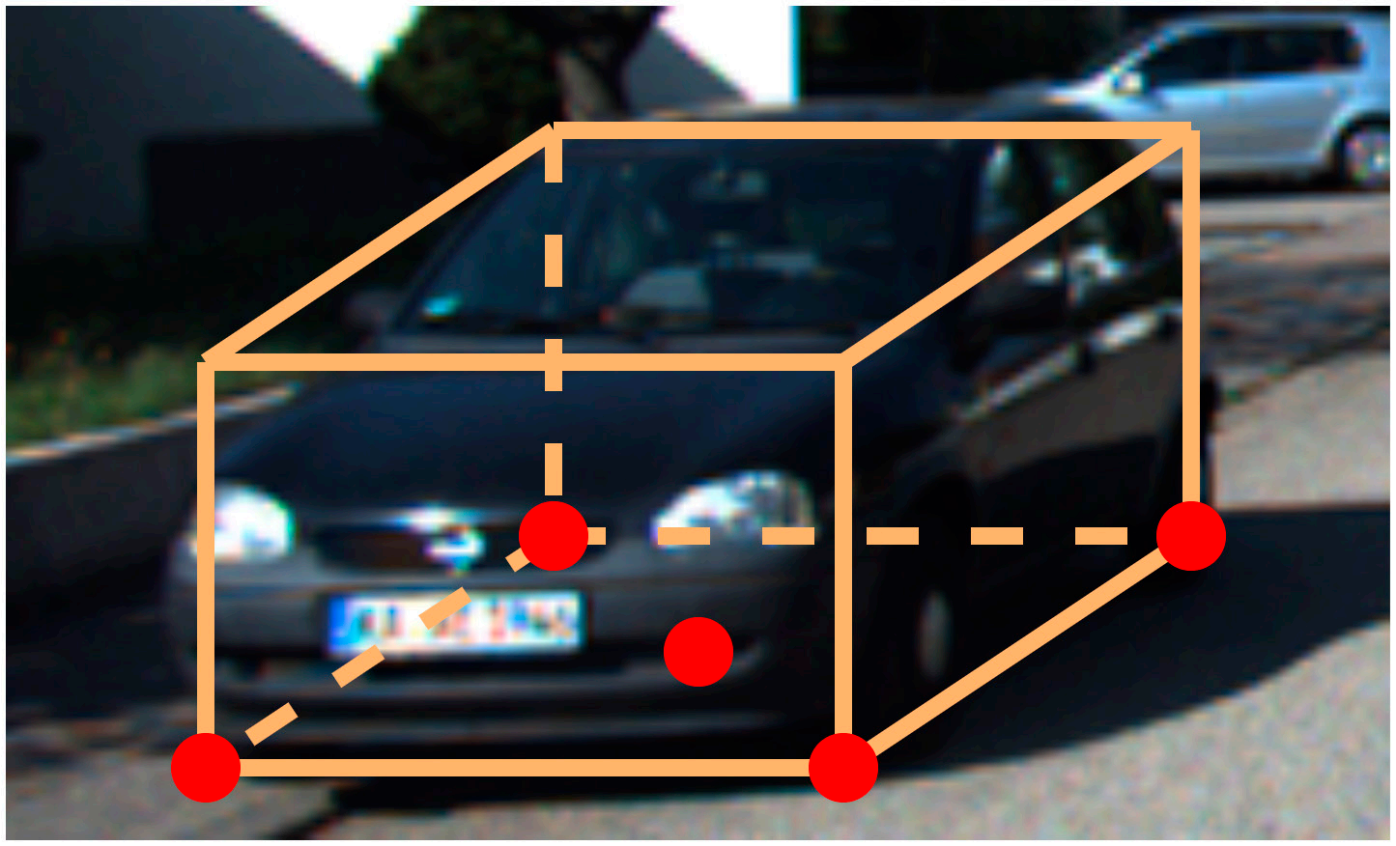
Five candidate points of a single object.

Given that the projection of objects onto the BEV plane does not exhibit intersections, it inherently contains additional 3D positioning information. Consequently, the homography matrix *M* is defined to relate the image plane and the BEV plane, implicitly facilitating the conversion of coordinates from a 2D to a 3D space. As illustrated in Fig. 2, we pick up five bottom points *Q_pred_* = [*x_pred_*, *y_pred_*, *z_pred_*]^T^** of predicted box as representatives [16]. These key points encompass the four corner points and the center of the bottom plane. Thus, for *N* objects, 5*N* pairs of candidate points for loss computation are needed.

More specifically, for each point pair, the ground truth coordinates, denoted as *q_gt_*, in the 2D image view candidate point are employed as a reference to refine the ultimate 3D spatial position:

$$Q_{\text{pred}}={\mathrm{Mq}}_{\mathrm{gt}}$$ (2)

where *M* encapsulates the interrelationships among all objects through the mapping between two views. Previously, 3D detection is constrained by regression loss, such as *L_reg_* = *L*1(*Q_gt_* − *Q_pred_*), where *Q_gt_* = [*x_gt_*, *y_gt_*, *z_gt_*]^T^**. Different from before, employing 2D ground truth projection points along with *M* to constrain 3D predictions results in the following formulation for the added homography loss:

Lhomo=SmoothL1Qgt−Qpred (3)

=SmoothL1Qpred−Mqgt (4)

As the training process progresses, the estimated value of *Q_pred_* gradually converges toward *Q_gt_* and *Mq_gt_* gradually converges toward *Q_pred_*. Calculating the homography matrix M considers all pairs of corresponding points, making it a global geometric constraint. By optimizing the loss in Eq. 3, *M* is also brought closer to the ground truth homography matrix. Consequently, this geometrically constrained global loss serves as a guiding mechanism for predicting 3D positions from ground truth 2D positions.

#### Occlusion head

In autonomous driving, it is common for targets to experience varying degrees of occlusion [36]. To enhance the precision of 3D object estimation, we propose integrating an occlusion branch for classifying occlusion levels, thereby providing more supervisory information for detected objects. Given the inherent challenge in accurately inferring occluded objects, it becomes crucial to incorporate the occlusion level into the model’s learning process, as it potentially influences the prediction of the object’s position and associated uncertainty [37].

The degree of target occlusion is categorized into *N* distinct levels. Among these levels, 0, 1, ..., *N* − 1 denote increasing degrees of object occlusion, with higher levels signifying greater occlusion extents. Level *N* indicates that the degree of occlusion remains unknown. We treat this as a multi-classification task and employ the focal loss [38]. The ground truth values for occlusion levels are derived from the dataset’s annotation information.

#### Keypoint offset head

Monocular 3D target detection tasks typically involve 2D detection as a foundational aspect. Recognizing the pivotal role of robust 2D detection in enhancing the performance of 3D detection tasks, a wealth of object information can be gleaned from the annotated 3D bounding boxes projected onto the 2D plane. Given the annotated 3D bounding box information and the object’s camera matrix in the image, we can derive the object’s projection details onto the image plane [39]. This information enables the calculation of offset vectors from the eight projected corners of the 3D bounding box to the center of the 2D bounding box. Incorporating the 2D projection data from the corner points of the 3D bounding box introduces additional constraints that enhance the overall detection task, and we apply the smooth-L1 loss [40] for this purpose.

#### 2D size head

In the 2D detection head of the 3D detection task, most detectors presently predict the lower-left and upper-right corner points of the 2D bounding box to reconstruct the 2D bounding box [41,42]. Recognizing that the dimensions of 2D bounding boxes contain valuable geometric information, we advocate for additional supervision in this regard. We propose the 2D dimensions branch to furnish more supervisory information for the holistic detection task.

### Uncertainty-guided 3D object depth and confidence

#### Diverse depth estimations

In this work, we address the issue of excessive error in monocular 3D target detection by proposing a depth fusion system. Unlike existing approaches, our system leverages auxiliary information to generate multiple estimates and captures their associated uncertainties. It then integrates these estimates through uncertainty-based fusion to yield a final result. Uncertainty-based depth fusion ensures that depth values with higher confidence receive more weight in the fusion process, enhancing accuracy while remaining robust to potentially inaccurate depth estimation.

Given knowledge of the camera matrix, the relative ratio between the pixel height (*h*) and the estimated object height (*H*) can be employed for determining the object’s depth (*z*) via:

$$z=f*\frac{H}{h}$$ (5)

where *f* represents the camera’s focal length. Solving for depth from height offers the advantage of being independent of orientation estimation and less susceptible to size estimation errors, as demonstrated in [29]. Based on the prior knowledge, we propose three additional sets of depth values derived from height to complement the baseline model.

**Fig. 3.**
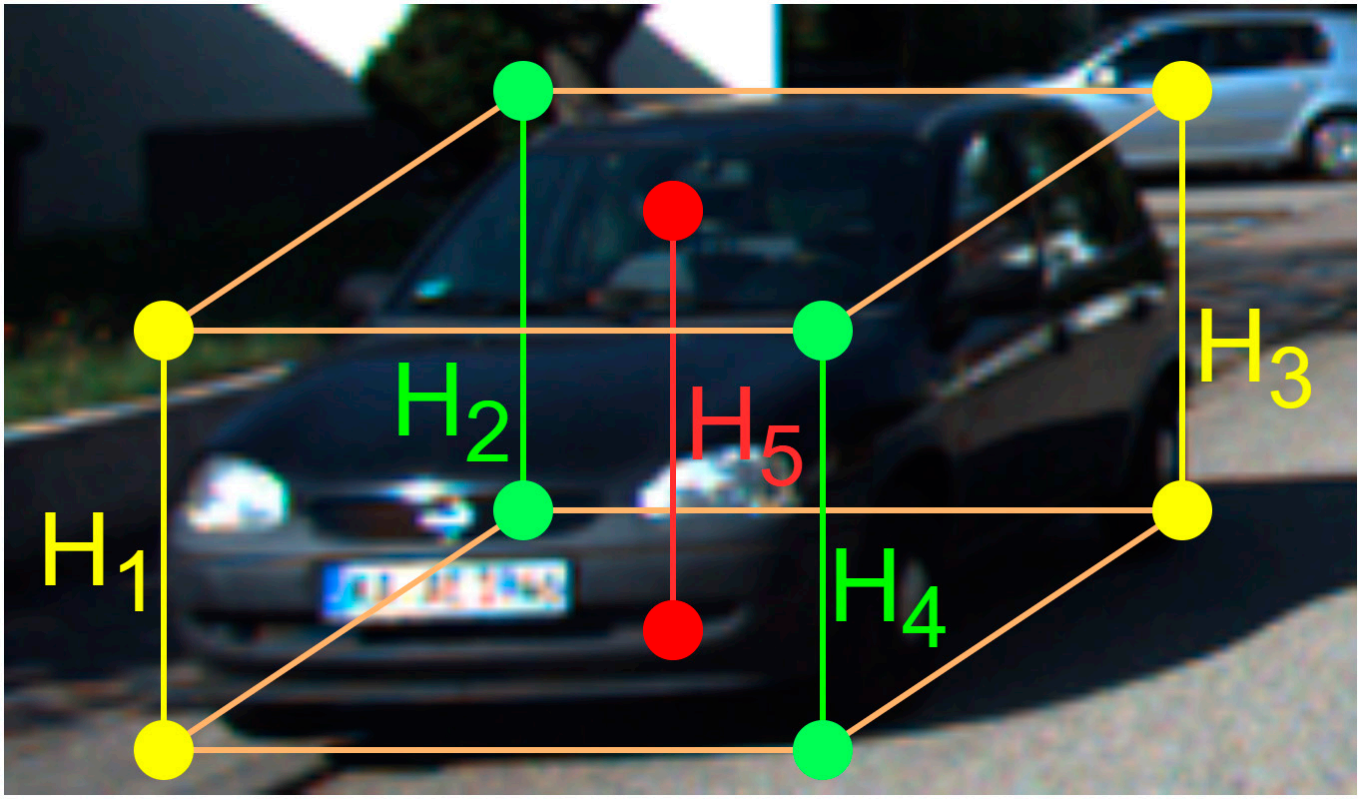
Depths from keypoints.

As illustrated in Fig. 3, the heights of the central vertical line and corner vertical lines are categorized into three groups: [*H*_1_, *H*_3_], [*H*_2_, *H*_4_], and [*H*_5_]. The object’s depth can be computed using the central vertical line height *H*_5_ and Eq. 5, or alternatively by averaging the depths generated using corner vertical lines, [*H*_1_, *H*_3_], [*H*_2_, *H*_4_]. Consequently, three groups of independently estimated depth values are obtained: the central depth, denoted as *z_c_*, along with two corner depth values, denoted as *z*_*d*1_ and *z*_*d*2_, respectively.

To guide the computation of keypoint depths and model their associated uncertainty, an L1 loss function with awareness of uncertainty [31] is employed, as follows:

$$\begin{array}{c} L_{\mathrm{kpd}}=\sum_{k\;\in \;\left\{c,d1,d2\right\}}\left[\frac{\left|z_{k}-z^{*}\right|}{\sigma_{k}}+\log\left(\sigma_{k}\right)\right] \end{array}$$ (6)

where *z_k_* represents the estimated depth value, *z*^∗^ signifies the ground truth value, and *σ_k_* represents the heteroscedastic aleatoric uncertainty.

Moreover, the baseline model uses a multivariate laplace distribution to jointly model physical and visual heights solved for a set of depth values [43], using the product of the elements on the diagonal of their covariance matrix as the uncertainty for that set of depths.

With four groups of depth values and their corresponding uncertainties mentioned above, the uncertainty information is harnessed to calculate a weighted average for the depths. Let *N* = 4, the weight assigned to each group’s uncertainty is determined by:

$$\omega_{i}=\frac{1/\sigma_{i}^{2}}{\sum_{j=1}^{N}\;1/\sigma_{j}^{2}}$$ (7)

Finally, the ultimate depth value and its associated uncertainty are expressed as follows:

$$z_{d}=\sum_{i=1}^{N}\;\omega_{i}z_{i}$$ (8)

$$\sigma_{d}^{2}=\sum_{i=1}^{N}\;\omega_{i}^{2}\sigma_{i}^{2}$$ (9)

#### Uncertainty-guided 3D object confidence

Let *P* denote the probability of accurately detecting a target, often referred to as “confidence.” In accordance with the probability chain rule outlined in [44], this probability can be decomposed into two components:

$$P=P_{2d}\cdot P_{3d|2d}$$ (10)

where *P*_2*d*_ is represented by the classification score in the 2D task, while *P*_3*d*∣2*d*_ signifies the conditional 3D confidence. Previous work [45] has explored the use of 3D intersection over union (IOU) to model *P*_3*d*∣2*d*_ . However, a common challenge arises in practical scenarios where only training images are available during the training process, and the images in the validation set are unknown. Typically, the average 3D box IOU of the model on the training set significantly exceeds that on the validation set. Consequently, directly employing 3D IOU for training and treating the predicted 3D IOU as *P*_3*d*∣2*d*_ yield suboptimal results in the validation phase.

Furthermore, prior research suggests that models capable of learning uncertainty through implicit supervision tend to exhibit superior generalization capabilities. Therefore, we choose to model *P*_3*d*∣2*d*_ in terms of implicitly acquired heteroscedastic uncertainty within the loss function. Specifically, uncertainty in each of the 3D branches, including depth, projection center point, dimensions, and yaw, is taken into account, and a single uncertainty value denoted as *P_u_* is derived through the fusion process outlined in Eq. 8.

The reciprocal of this uncertainty, denoted as 1/*P_u_*, is utilized as *P*_3*d*∣2*d*_, which is then combined with *P*_2*d*_ to formulate the final confidence, denoted as *P_m_*:

$$P_{m}=\left(1/P_{u}\right)\cdot P_{2d}$$ (11)

### Universal multi-task training

Considering the challenge of adjusting inter-task weights when adding branches, we propose a multi-task learning strategy based on progressive training. This strategy proposes improvements in data preprocessing and loss optimization, and it can adjust loss weights adaptively.

#### Training samples pre-processing

In contrast to prevailing approaches that prioritize the emphasis on learning hard samples [46] , we suggest disregarding exceptionally hard samples due to their potential to lead to misguidance in the model’s learning process.

Objects situated at significant distances within monocular images occupy very few pixels on the imaging plane. The inclusion of such samples in the training dataset may introduce inaccuracies into the model’s overall learning process. In particular, we exclude targets located at a distance of 60 m or more when they are also occluded:

$$\omega_{i}=\left\{\begin{array}{ll} 1 & \mathrm{if}\;\;d_{i}\leq 60\\ 0 & \mathrm{if}\;\;d_{i}>60\;\mathrm{and}\;\mathrm{occluded} \end{array}\right.$$ (12)

where *w_i_* represents the object-level training weight assigned to sample *i* and *d_i_* denotes the actual distance of the sample.

It is noteworthy that these excluded samples constitute only a small fraction of the entire training dataset, and their removal does not impact the network’s representation learning across the broader dataset.

#### Scale-aware L1 loss

In the context of monocular 3D detection, the task of depth estimation is inherently ill-posed. It is challenging to recover the depth of a target from a monocular image, leading the model to emphasize depth optimization. Consequently, the contribution of the depth branch to the overall task learning frequently overshadows that of other branches. Decoupling each loss term and optimizing them independently will result in significant performance degeneration due to the lack meaningful interconnections.

The relationship between IOU and the partial derivative of size corresponds to the reciprocal of the size parameter [46]. Accordingly, the weight of each side can be adjusted based on its partial derivative with respect to IOU. Therefore, we propose an adaptive scale-aware L1 loss for both the 2D size and 3D size branches in the detection task:

$$L_{\text{size}}={\left\|\frac{\left(s-s^{*}\right)}{s}\right\|}_{1}$$ (13)

where ‖⋅‖_1_ represents the L1 norm and *s* represents [*h*, *w*, *l*]_3*D*_ or [*h*, *w*]_2*D*_. This loss function can be conceptualized as a redistribution of the conventional L1 loss.

#### Progressive training strategy

In the “Diverse depth estimations” section, we address the issue of excessive error in monocular 3D target detection by proposing a depth fusion system, However, while this approach has its advantages, it can also introduce challenges during the training process. Specifically, at the onset of training, predictions of both visual and physical heights are typically imprecise, potentially misleading the overall training process and adversely affecting performance. Moreover, the incorporation of multiple auxiliary branches within the module can exert varying degrees of influence on the primary detection branch. Consequently, it becomes imperative to employ suitable strategies to adjust the relative importance of each branch and its contribution to the overall loss.

In order to solve this problem, we adopt a progressive training strategy, which dynamically adjusts the weight of each branch loss in different training stages:

$$L_{\mathrm{sum}}=\sum_{i\in S}\;\omega_{i}\left(t\right)\cdot L_{i}$$ (14)

where *S* is the task set, *t* represents the current iteration index, and *L_i_* represents the *i*-th task loss function. *ω_i_*(*t*) is the loss weight of the *i*-th task in round *t*.

**Fig. 4.**
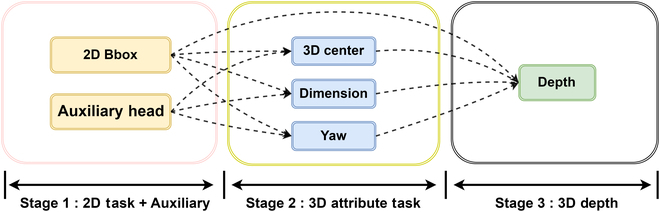
The proposed progressive training strategy.

It is noteworthy that each task should undergo training only after its preceding tasks have been adequately trained. This process is systematically divided into distinct stages, as illustrated in Fig. 4.

The weight for the *i*-th loss term *ω_i_*(*t*) should be associated with all predecessor tasks of the *i*-th task. The initial stage encompasses tasks related to 2D detection and the supplementary branches we have proposed. The second stage incorporates the 3D attribute training, encompassing aspects such as angles and 3D dimensions. The final stage presents the most challenging aspect, which is depth inference. This stage is dependent on the successful completion of all tasks within the 3D dimension and 2D detection stages.

To facilitate a gradual increase in the weight parameter, *ω_i_*(*t*), from 0 to 1 during training, we employ a polynomial time scheduling function [47]:

$$\begin{array}{c} \omega_{i}\left(t\right)={\left(\frac{t}{T}\right)}^{1-\alpha_{i}\left(t\right)}\;\;,\;\;\alpha_{i}\left(t\right)\in \left[0,1\right]\; \end{array}$$ (15)

where *T* is the total training period and the normalized time variable *t*/*T* can automatically adjust the time scale. *α_i_*(*t*) is the adjustment parameter of the *t*-th iteration, corresponding to each predecessor task of the *i*-th task, where *α_i_*(*t*) is expressed as:

$$\alpha_{i}\left(t\right)=\prod_{\;j\in G_{i}}\;S_{j}\left(t\right)$$ (16)

where *G_i_* is the set of predecessor tasks of the *i*-th task. *S_j_* represents the learning status indicator of the *j*-th task, which is a value between 0 and 1. For *S_j_*, inspired by [19], we use scale-invariant factors to indicate the learning situation:

$$S_{j}\left(t\right)=\frac{|{\mathrm{LS}}_{j}\left(K\right)-{\mathrm{LS}}_{j}\left(t\right)|}{{\mathrm{LS}}_{j}\left(K\right)}$$ (17)

$${\mathrm{LS}}_{j}\left(t\right)=\frac{1}{K}\sum_{t_{k}=t-K}^{t-1}\left|L_{j}\left(t_{k}\right)\right|\;$$ (18)

where *L_j_*(*t_k_*) represents the derivative of *L_j_*(⋅) at the *t_k_*-th iteration, serving as an indicator of the local change trend in the loss function. *LS_j_*(*t*) computes the average derivative over the most recent *K* iterations leading up to the *t*-th iteration, capturing the trend in mean changes. The *S_j_* formula is employed to compare the current trend represented by *LS_j_*(*t*) with the trend observed in the preceding *K* iterations when training for the *j*-th task begins *LS_j_*(*K*). If the current loss trend closely aligns with the initial trend, this indicator yields a smaller value, suggesting that the task’s training has not been sufficiently effective.

This approach is proposed based on an overall design in which the weight loss of each component can dynamically reflect the learning status of its predecessor tasks.

## Results

The proposed framework is primarily evaluated using the KITTI 3D Object Detection benchmark [21], which comprises various challenges tailored for computer vision in the context of autonomous driving. This dataset comprises 7,481 images for training and 7,518 images designated for testing purposes. To maintain consistency with the [48] approach, we follow their protocol to partition the training images into training and validation sets, comprising 3,712 and 3,769 images, respectively.

The assessment of detection results is conducted across three distinct difficulty levels: easy, moderate, and hard. These levels are defined based on criteria such as bounding box size, occlusion, and truncation. It is important to note that all reported results are generated by models that simultaneously detect multiple classes, including cars, pedestrians, and cyclists. Additionally, we will present the KITTI BEV benchmark detection results for reference.

Moreover, we utilized the Waymo dataset [22] to evaluate our approach. It offers 798 training sequences and 202 validation sequences from diverse scenarios, making it a comprehensive and challenging dataset for autonomous driving. Adhering to the methodology outlined in the baseline model [43], we constructed our training set (52,386 images) by sampling every third frame from the 798 training sequences and formed our validation set (39,848 images) by using all frames from the 202 validation sequences. Our model evaluation is conducted based on the vehicle category.

### Evaluation metrics

For the KITTI dataset [21], the primary metrics employed for performance comparison are the AP computed for 3D bounding boxes and BEV maps. Specifically, for the validation set, we provide the *AP*3*D* ∣ *R*40 metric to facilitate a comprehensive comparison with previous research studies. In this context, the IOU threshold for AP3D is set at 0.7 for cars.

For the Waymo dataset [22], we assessed the *AP3D* metric at various IOU thresholds and categorized the results into two levels, namely, *LEVEL*_1 and *LEVEL*_2, based on the detection difficulty.

### Implementation details

We adopt MonoRCNN++ [43] as our baseline architecture, employing a backbone composed of ResNet-50 [49], which is pre-trained on the ImageNet dataset [50]. For region-of-interest (ROI) feature extraction, we utilize feature maps (size: 256 × 7 × 7) from the P2, P3, P4, and P5 levels of the backbone network, as prescribed in [35].

Our anchor configuration comprises five scale anchors 32, 64, 128, 256, and 512 and three aspect ratios 0.5, 1, and 2. Both the core detection head within the fundamental framework and the auxiliary branch introduced later consist of two hidden fully connected layers (size: 1,024) followed by an output fully connected layer.

The model undergoes training on 4 RTX 2080Ti GPUs, with a total of 120,000 iterations. Our training batch size is set to 8. During the training process, we apply random horizontal mirroring and photometric distortion as data augmentation techniques, while no augmentation is employed during inference. Implementation-wise, our approach is realized using the PyTorch framework [51] and the Detectron2 library [52].

### Quantitative results

Table 1 presents a comprehensive comparison between our proposed method and the state-of-the-art techniques across three categories within the validation set of the KITTI benchmark [21]. Our method demonstrates superior performance when compared to most approaches relying solely on monocular images as input, and it remains competitive among methods incorporating additional input modalities. Notably, our method exhibits notable improvements over MonoRCNN++ [43].

**Table 1.** Quantitative results for Car on KITTI val sets [21]. “Input” means the input data modality used during training. We divide existing methods into two groups considering whether they utilize extra information. * means we use the retrained results from the official open-source code, which are slightly different from the reported results.

Method	Input	Times(ms)	*AP*_3*D*_, *IoU* ≥ 0.7[Easy/Mod/Hard]↑	*AP_BEV_*, *IoU* ≥ 0.7[Easy/Mod/Hard]↑
AM3D(ICCV’19)[54]	Image + Depth	400	28.31/15.76/12.24	25.03/17.32/14.91
ForeSeE(AAAI’20)[55]	Image + Depth	-	15.00/12.50/12.00	23.40/17.40/15.90
UR3D(ECCV’20)[56]	Image + Depth	120	23.24/13.35/10.15	- / - / -
Kinem3D(ECCV’20)[57]	Image + Video	120	19.76/14.10/10.47	26.69/17.52/13.10
D4LCN(CVPR’20)[58]	Image + Depth	-	22.32/16.20/12.30	22.51/16.02/12.55
CaDDN*(CVPR’21)[25]	Image + Depth	40	22.17/15.84/12.77	- / - / -
M3D-RPN(ICCV’19)[59]	Image	160	14.53/11.07/8.65	25.94/21.18/17.90
MonoDIS(CVPR’19)[60]	Image	100	18.05/14.98/13.42	24.26/18.43/16.95
Movi3D(ECCV’20)[61]	Image	45	14.28/11.13/9.68	22.36/17.87/15.73
MonoPair(CVPR’20)[12]	Image	60	16.28/12.30/10.42	24.12/18.17/15.76
MonoRCNN(ICCV’21)[14]	Image	70	16.61/13.19/10.65	25.29/19.22/15.30
MonoDLE(CVPR’21)[46]	Image	40	17.45/13.66/11.68	24.79/18.89/16.00
GrooMeD(CVPR’21)[62]	Image	120	19.67/14.32/11.27	26.19/18.27/14.05
MonoGeo( - )[63]	Image	50	18.45/14.48/12.87	27.15/21.17/18.35
AutoShape(ICCV’21)[64]	Image	50	20.09/14.65/12.07	- / - / -
MonoGround*(CVPR’22)[30]	Image	30	20.54/15.27/**13.51**	28.78/21.51/17.85
DID-M3D*(ECCV’22)[15]	Image	40	20.18/15.39/12.03	**30.03**/21.26/18.13
Edge-RCNN(WACV’23)[17]	Image	-	18.44/14.60/12.57	26.19/20.67/17.30
M-RCNN++(WACV’23)[43]	Image	70	19.07/14.87/12.59	26.41/20.80/17.27
MonoAux(ours)	Image	70	**20.58**/**15.64**/12.96	29.13/**21.77**/**18.44**

For the *AP*3*D* ∣ *R*40 metric, focusing on the car category within the validation set, our method showcases impressive enhancements, registering increases of 7.9%, 5.1%, and 3.1% across three different difficulty levels. Similarly, concerning the *APBEV* ∣ *R*40 indicator, we observe substantial improvements of 10.2%, 4.7%, and 6.8% across the same three difficulty levels.

The overarching improvement across all difficulty levels underscores our method’s effectiveness, not only in enhancing overall performance on simpler and moderately challenging scenarios but also in addressing the complexities posed by occluded or truncated objects. Moreover, our method surpasses most existing techniques in terms of speed, enabling real-time inference—a crucial feature for ensuring real-time safety in autonomous driving applications. For clarity, we highlight the best-performing methods that solely employ images as input in bold font.

Subsequently, we conducted benchmark testing on our method using the Waymo Val split [22], shown in Table 2. For ease of comparison, we provide the baseline model results retrained in the same environment. When the IOU threshold is 0.7, our approach demonstrates a comprehensive improvement compared to the baseline. It outperforms the second-ranking method across all levels, achieving the best performance in all evaluated metrics. Particularly noteworthy is its substantial lead over the second-ranked method, surpassing DEVIANT [53] by 45.72%/41.67% at LEVEL 1/LEVEL 2. Even at an IOU threshold of 0.5, our approach maintains a substantial improvement over the baseline. However, in comparison to the DEVIANT [53] method, it does not achieve the state-of-the-art performance. Our overall AP scores at LEVEL 1 and LEVEL 2 respectively secure the second and third positions when compared to DEVIANT [53].

**Table 2.** Quantitative results for vehicle on Waymo val sets [23]. “Input” means the input data modality used during training. “I” denotes image, and “D” denotes depth. * means we use the retrained results from the official open-source code, which are slightly different from the reported results.

Method	Input	LEVEL_1(*AP*_3*D*_, *IoU* > 0.5)↑				LEVEL_2(*AP*_3*D*_, *IoU* > 0.5)↑			
Overall		0-30m	30-50m	50m+	Overall	0-30m	30-50m	50m+
PatchNet(ECCV’20)[23]	I+D	2.92	10.03	1.09	0.23	2.42	10.01	1.07	0.22
PCT(NeurIPS’21)[65]	I+D	4.20	14.70	1.78	0.39	4.03	14.67	1.74	0.36
M3D-RPN(ICCV’19)[59]	I	3.79	11.14	2.16	0.26	3.61	11.12	2.12	0.24
GUPNet(ICCV’21)[19]	I	10.02	24.78	4.84	0.22	9.39	24.69	4.67	0.19
MonoJSG(CVPR’22)[28]	I	5.65	20.86	3.91	**0.97**	5.34	20.79	3.79	**0.85**
DEVIANT(ECCV’22)[53]	I	**10.98**	**26.85**	**5.13**	0.18	**10.29**	**26.75**	**4.95**	0.16
M-RCNN++(WACV’23)[43]	I	7.14	21.71	3.17	0.33	6.69	21.63	3.06	0.29
MonoAux(ours)	I	9.82	25.15	3.89	0.38	9.25	25.07	3.78	0.35
Method	Input	LEVEL_1(*AP*_3*D*_, *IoU* > 0.7)↑				LEVEL_2(*AP*_3*D*_, *IoU* > 0.7)↑			
Overall		0-30m	30-50m	50m+	Overall	0-30m	30-50m	50m+
PatchNet(ECCV’20)[23]	I+D	0.39	1.67	0.13	0.03	0.38	1.67	0.13	0.03
PCT(NeurIPS’21)[65]	I+D	0.89	3.18	0.27	0.07	0.66	3.18	0.27	0.07
M3D-RPN(ICCV’19)[59]	I	0.35	1.12	0.18	0.02	0.33	1.12	0.18	0.02
GUPNet(ICCV’21)[19]	I	2.28	6.15	0.81	0.03	2.14	6.13	0.78	0.02
MonoJSG(CVPR’22)[28]	I	0.97	4.65	0.55	0.10	0.91	4.64	0.55	0.09
DEVIANT(ECCV’22)[53]	I	2.69	6.95	0.99	0.02	2.52	6.93	0.95	0.02
M-RCNN++*(WACV’23)[43]	I	1.71	6.12	0.50	0.04	1.60	6.10	0.48	0.03
MonoAux(ours)	I	**3.92**	**10.29**	**1.34**	**0.23**	**3.57**	**10.13**	**1.26**	**0.19**

We attribute this discrepancy to DEVIANT’s utilization of scale data augmentation during training, enhancing model accuracy and resulting in superior performance under less strict evaluation (IOU > 0.5). The more stringent evaluation criteria (IOU > 0.7) demand higher overlap, allowing our model to more accurately localize target positions. This advantage is likely attributable to the additional information supervision and introduced uncertainty in our approach. Also, for clarity, we highlight the best-performing methods in bold font.

### Ablation study

#### Auxiliary regression heads

In Table 3, a comparative analysis is conducted to assess the influence of supplementary auxiliary regression heads, which encompass the homography loss head, occlusion head, key point offset head, and 2D size head. The experiment results presented here and later correspond to the indicators of the car category on the KITTI validation set [21]. Experiment (a) uses a baseline manually trained on 4 RTX 2080Ti GPUs.

Our observations are as follows: (a) The incorporation of auxiliary regression branches yields performance enhancements when compared to the baseline, underscoring the essential nature of additional supervision in the context of monocular 3D object detection. (b) Notably, the homography branch introduces constraints by leveraging the geometric relationship between the BEV plane and the 2D projection plane. This constraint mechanism enriches spatial information and results in more substantial improvements compared to other branches that confine constraints solely within the 2D projection plane. (c) The efficacy of adding supplementary auxiliary heads is consistent across easy, moderate, and hard samples. This reaffirms the notion that the inherent information available in the monocular 3D detection task is indeed limited, necessitating the introduction of additional informative constraints.

#### Uncertainty-guided 3D object depth and confidence

In Table 4, we evaluate the impact of introducing the uncertainty-guided 3D object depth and confidence policy. The experiment (f) is a modified baseline model that operates without any uncertainty consideration and provides only a single depth value.

**Table 3.** Ablation study on auxiliary regression heads

	Occlusion	2D size	kpt offset	Homography	*AP*_3*D*_|_*R*40_[Easy/Mod/Hard]
(a)					18.15/13.67/11.60
(b)	✓				18.39/13.90/11.90
(c)	✓	✓			18.71/13.98/12.37
(d)	✓	✓	✓		18.57/14.23/12.12
(e)	✓	✓	✓	✓	18.99/14.66/12.33

Through experiments denoted as (f) and (g), we observed that instructing the model to generate multiple sets of depth values yields superior results when compared to relying on a single depth value. This approach not only enhances the overall performance but also mitigates errors inherent to a single depth estimation. Furthermore, the integration of depth guided by uncertainty proves notably more effective than the simple average strategy for obtaining the final depth value. Our approach yields depth estimates that are more reliable and precise.

The incorporation of uncertainty-corrected 3D confidence into the model results in significant improvements over the baseline. This observation underscores the capacity of uncertainty information to assist the model in better distinguishing between accurate and inaccurate target identifications.

#### Universal multi-task training

In Table 5, we conduct a comparative analysis to assess the impact of the UMT strategy and its individual components. The experiment (k) is our baseline, which has been augmented with auxiliary branches and modified to incorporate the uncertainty-guided 3D object depth and confidence policy.

**Table 4.** Ablation study on uncertainty-guided 3D object depth and confidence. “AD” means using average 3D object depth. “UD” means using uncertainty-guided 3D object depth. “UC” means using uncertainty-guided 3D object confidence.

	AD	UD	UC	*AP*_3*D*_|_*R*40_[Easy/Mod/Hard]
(f)				16.93/12.27/10.52
(g)	✓			17.35/12.64/10.71
(h)		✓		17.71/12.96/10.99
(i)			✓	18.15/13.67/11.60
(j)		✓	✓	19.35/14.38/12.21

**Table 5.** Ablation study on uncertainty-guided 3D object depth and confidence. “PreP” means process training samples. “S-L1” means using scale-aware L1 loss instead of L1 loss. “Pro-train” means using progressive training strategy.

	PreP	S-L1	Pro-train	*AP*_3*D*_|_*R*40_[Easy/Mod/Hard]
(k)				19.71/14.86/12.36
(l)	✓			19.88/14.99/12.48
(m)	✓	✓		20.17/15.14/12.41
(n)	✓	✓	✓	20.58/15.64/12.96

Our experimental observations reveal the following insights: pre-processing of sample data that are excessively challenging prior to training initiates slight improvements. The exclusion of such data, constituting only a minor fraction of the overall dataset, does not significantly hinder the model’s overall learning process. Instead, it contributes to a modest enhancement. The inclusion of the scale-aware L1 loss allows the model to acquire more comprehensive and efficient learning of target sizes. This component facilitates a more detailed understanding of the target size information. The progressive training strategy proves effective in preventing the model from succumbing to early training misdirection, thereby enhancing training stability. Our proposed approach ensures that the model’s initial learning phases are more reliable. The amalgamation of the UMT strategy yields discernible improvements when applied to the original model. This outcome substantiates the efficacy of the UMT strategy proposed in our work.

### Qualitative results

**Fig. 5.**
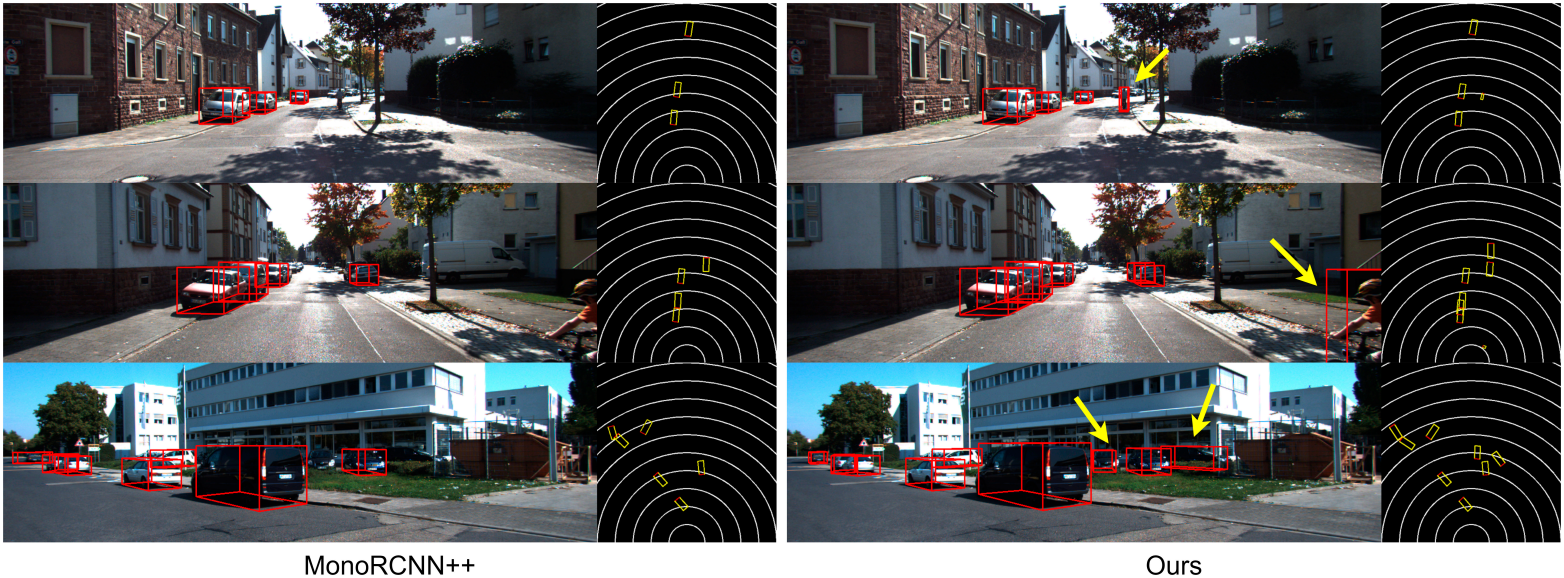
Qualitative results. We visualize 3D object detection and BEV detection results on KITTI val set [21]. Left: The detection results of MonoRCNN++. Right: The detection results of our proposed MonoAux. Comparative differences are pointed out with yellow arrows.

**Fig. 6.**
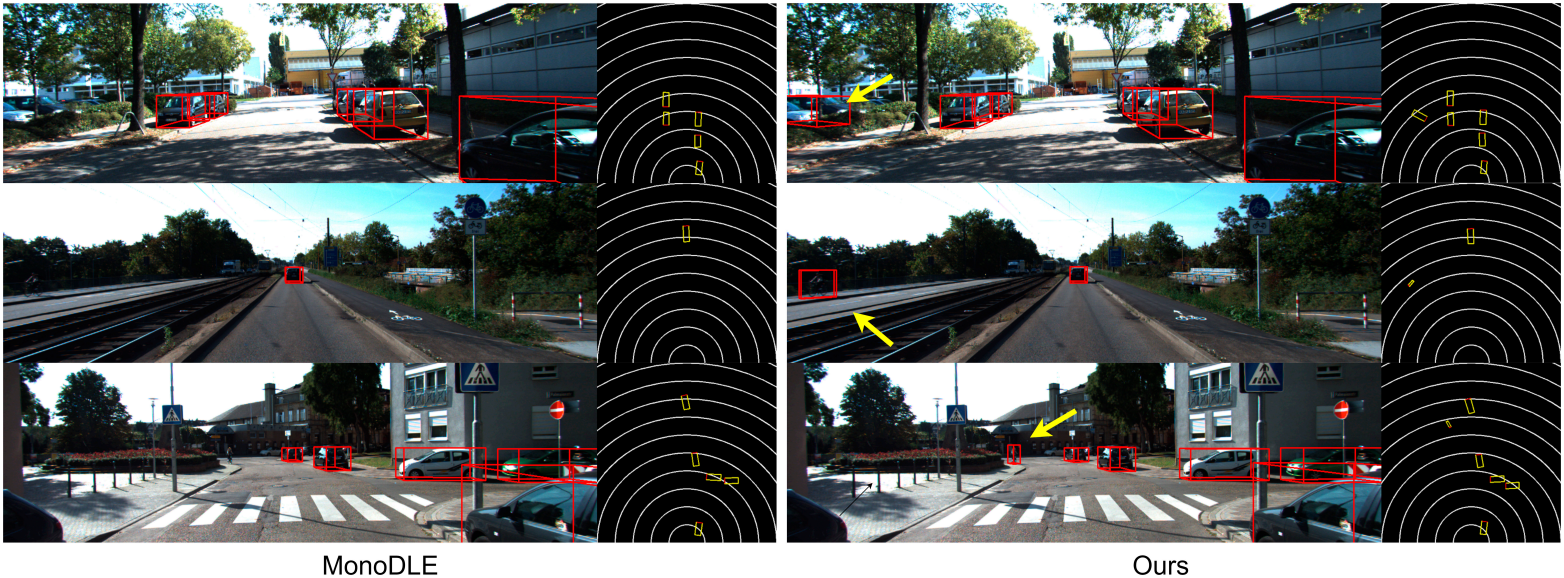
Qualitative results. Qualitative results of MonoDLE and our method.

**Fig. 7.**
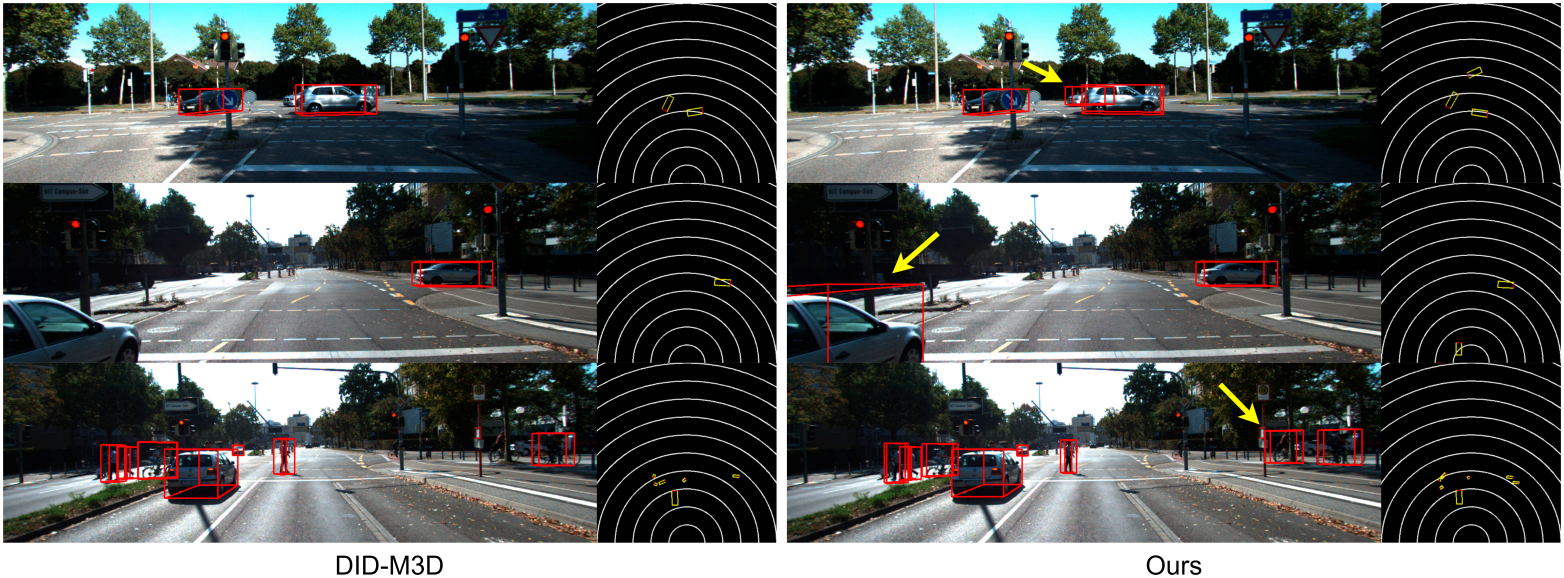
Qualitative results. Qualitative results of DID-M3D and our method.

From the qualitative results shown in Figs. 5 to 7, our proposed framework demonstrates superior performance in effectively identifying common objects within diverse street scenes. To facilitate comparison, we have standardized the display format of the visualization results. The performance disparity between the proposed framework and other models is illustrated. Certainly, our model demonstrates notable detection performance compared to other models for small target objects, occluded entities, and normal objects that may elude detection. This is evident in both the 2D image perspective and BEV perspective, substantiating the efficacy of our proposed module in effectively handling detection across a majority of scenarios.

## Discussion

In this work, we propose a monocular 3D object detection framework emphasizing enhanced information utilization, diverse depth estimation, and integration of uncertainty considerations. Leveraging implicit information in 3D annotations, we establish transformation relationships between planes to address data scarcity in monocular 3D detection. Our approach uses estimated uncertainty to dynamically combine depth values and correct 3D confidence, reducing errors in individual detection results. Additionally, we propose a flexible training strategy for multi-task learning in monocular 3D target detection, targeting inaccuracies in position and size estimation during training.

The evaluation on the KITTI and Waymo benchmark demonstrates the comprehensive improvements achieved by our method compared to the original framework. Our findings underscore the significance of introducing additional information and embracing uncertainty considerations in object detection, particularly in the challenging context of monocular 3D object detection.

Nevertheless, there are limitations in this work. The inclusion of supervisory information during training significantly extends the model’s training time. Subsequent to the introduction of uncertainty, both the fused depth value and the adjusted 3D confidence level hinge on accurate uncertainty estimates. While this contributes to an overall enhancement in the model’s performance, it cannot be discounted that a small subset of samples, when subjected to imprecise uncertainty estimation, may yield inferior detection results compared to their original counterparts. Therefore, exploring more accurate methods for uncertainty estimation will be a crucial direction for future research.

## Data Availability

Data used in this study is publicly available.
